# Does solo publication still make sense? Single-authored publications have been essential to scientific progress but are now facing extinction

**DOI:** 10.1038/s44319-025-00677-1

**Published:** 2025-12-16

**Authors:** Valentí Rull

**Affiliations:** 1 Botanic Institute of Barcelona, Spanish National Research Council (CSIC), Barcelona, Spain; 2 Institut Català de Paleontologia Miquel Crusafont (ICP-CERCA), Universitat Autònoma de Barcelona, Cerdanyola del Vallès, Spain

**Keywords:** History & Philosophy of Science, Science Policy & Publishing

## Abstract

Solo papers (SPs) have experienced a sustained decline over the last century, which has turned them into a residual publication type. However, SPs have traditionally been highly influential and disruptive, and efforts to protect them from extinction are worthwhile.

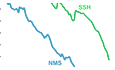

We have entered the fourth age of research, increasingly dominated by collaboration of research teams, often across borders (Adams, 2013). Since the beginning of this century, research teams have generated virtually all exceptionally high-impact papers across nearly all research fields, thereby displacing the earlier dominance of solo researchers (Wuchty et al, 2007). This shift has been described as the “death of the Renaissance man” (Jones, 2009), highlighting the growing impossibility for a single individual to cope with today’s research requirements and specialization. Persistent solo publishers are sometimes pejoratively labeled “lone wolves” (Lazebnick and Rosenfeld, 2025), referring to individuals who dislike working in groups or accepting others’ ideas because they perceive collaborators as less capable or effective (Barr et al, 2005). However, this is a biased and unwarranted oversimplification. It would be equally unfair to assume that scholars who never publish alone lack the ability to think independently, drawing an analogy with a “flocking sheep” stereotype.

“Persistent solo publishers are sometimes pejoratively labeled “lone wolves” […] referring to individuals who dislike working in groups…”

This paper describes how single-authored papers have declined over the last century and argues that these publications are nonetheless important to science and should be protected from extinction. It should be stressed that this is not a binary debate about the merits of multi-authored versus solo papers, which has been sufficiently discussed elsewhere (Wuchty et al, 2007; Praus, 2025). Rather, the focus here is the *raison d’être* of the latter in a world where the former has become the dominant publication.

## The decline

**Figure 1:**
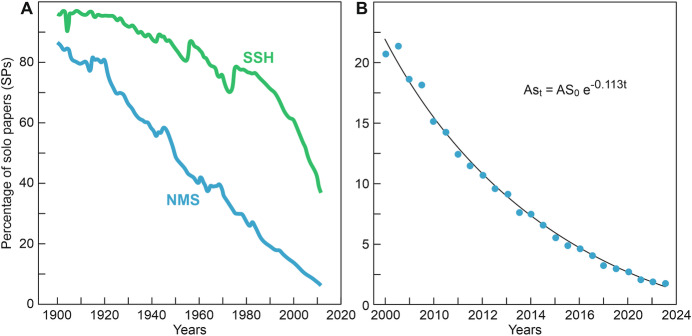
Historical decline of solo papers (SPs). (**A**) The trends in SP percentage in the natural and medical sciences (NMS) and the social sciences and humanities (SSH). Redrawn from Larivière et al (2015). (**B**) Decline in SP percentage during the last few decades in chemical engineering. Redrawn from Praus (2025).

The decline of solo papers (SPs) is evident. In 1900, 87% of natural and medical science (NMS) papers and 97% of social science and humanities (SSH) papers published were single-authored (Fig. 1A). By 2011, these numbers had fallen to 7% and 38%, respectively (Larivière et al, 2015). Interestingly, this decline is nearly linear in NMS, while in SSH has been slower with an acceleration since the 1980s. In both instances, extrapolating these general trends implies that solo publications would disappear soon. Nevertheless, this has not occurred and is unlikely to occur, as the decline in SPs during the past few decades (2000–2023) has followed an exponential function (Praus, 2025). In the Web of Science (WOS), from which the data used by Larivière et al (2015) were retrieved, coverage is strong for NMS but less extensive for SSH. Therefore, the trends observed in NMS can be considered more reliable, whereas those in SSH should be interpreted with greater caution.

This decline shows a good fit with exponential decay equations of the type SP_t_ = SP_0_ e^-kt^, where SP_t_ is the number of papers at time t, SP_0_ the initial number of papers and k the rate of decline (Fig. 1B). The highest rates of exponential decay are observed in chemical engineering, metallurgy and nanoscience (k = 0.075–0.113), with biology, environmental sciences and analytical chemistry showing intermediate values (k = 0.062–0.065). In contrast, sociology, law, history and theatre exhibit the lowest rates (k < 0.010) (Praus, 2025). According to this model, SPs will not disappear entirely but will remain as a residual publication type, and the process will be faster in the physicochemical and natural sciences than in the humanities and arts. However, in particular cases, the total extinction of SPs seems to be just around the corner. For example, in 2017, after a sustained decline, *Science* published only two SPs, and *Nature* did not publish any (Emmer, 2019).

I conducted a survey for the year 2024, which is not included in the above meta-analyses, using the publicly available OpenAlex database and obtained average values of 8% SPs for NMS and 40% for SSH. Among NMS, the highest rates were observed in physics and astronomy (14%), environmental sciences (13%) and neuroscience (11%), whereas the lowest values occurred in chemistry (5%), chemical engineering (5%) and materials science (4%). In SSH, the average percentage of SPs was significantly higher (40%), with the largest numbers in the arts and humanities (65%) and the lowest in psychology (24%). These recent data confirm that SPs remain still alive, being low in NMS and relatively high in SSH, as predicted by previous studies.

Under these conditions, it seems reasonable to ask whether SPs in NMS are still useful to science and worth continuing to publish. This would be equivalent to asking whether individual performance is still important in science, as it surely is in other activities such as art, literature or music—just to name a few—where individual authorship plays a central role. Or, in other words, whether the recent evolution of science toward team-oriented research has already passed a tipping point beyond which individual authors are no longer able to contribute given the complexity and specialization of many research topics.

“… it seems reasonable to ask whether SPs in NMS are still useful to science and worth continuing to publish.”

## Relevance of solo papers

It is trivial to point out that many major scientific discoveries and breakthrough advances—such as those made by Newton, Darwin or Einstein—were done by single individuals and published as SPs. Their contributions were largely the result of long-term observation, calculation and individual reflection, rather than complex experiments requiring sophisticated equipment, big data and statistical analysis software. This is not necessarily a strong argument in favor of SPs, since only a tiny proportion of “lone wolves” turn out to be geniuses of this kind. However, it may prompt reflection on what might have happened if these scholars had been required to work in teams, share their ideas and reach consensus before publication. The outcome might have been different.

The sustained decline of SPs recorded over the last century (Fig. 1) has had a greater impact on data papers—also known as original research papers—due to the increasing scale, specialization, complexity and cost of research, which often requires interdisciplinary and interinstitutional collaboration, and diverse methodological approaches (Wuchty et al, 2007). In contrast, theoretical and conceptual papers remain largely the domain of solo authorship. Currently, SPs are mostly theoretical in nature and require minimal collaboration, such as reviews, opinion pieces, essays, forum papers, short communications, invited contributions, lectures, letters to the editor, editorials or similar formats (Emmer, 2019; Praus, 2025). Data papers presenting novel results are only rarely published as SPs. However, this does not mean that SPs do not contribute to the advancement of knowledge. Scientific value lies not only in generating new data, but also in the ability to reassess existing evidence with new ideas and approaches, which can lead to paradigm shifts and open new lines of research. Theoretical papers are particularly well suited for this purpose.

“Scientific value lies not only in generating new data, but also in the ability to reassess existing evidence with new ideas and approaches, which can lead to paradigm shifts and open new lines of research.”

## Are solo papers more disruptive?

To analyze the impact of solo and team publications in terms of breakthrough capacity, Wu et al (2019), using Clarivate WOS data from 1954 to 2014, classified papers into two categories: developing and disruptive. The former are papers that build on existing and already successful scientific ideas, whereas the latter disrupt prior knowledge by revisiting older and less popular work or by exploring new ideas, thereby launching novel research avenues. These authors show that the disruptive capacity of papers is highest for SPs and progressively declines as the number of authors increases, with a notable drop after three authors. Moreover, the impact of SPs and two-author papers is more enduring and ultimately becomes more influential over time. These results are similar for both reviews and original research papers, indicating that it is the number of authors, rather than the article type, that makes the difference. Therefore, the number of authors seems to have a dilution effect on the disruptive capacity of papers.

In addition, it has been documented that the impact of team papers is more closely related to the lower-impact than to the higher-impact team members, known as the downgrading effect. As a consequence, there is a tendency for teams to be composed of members with similar impact levels (Ahmadpoor and Jones 2019). This may also lead high-impact researchers to avoid collaboration with certain groups: the escaping strategy. Finally, because credit allocation is difficult to assess in multi-authored papers—the credit homogenization effect—high-impact and highly creative researchers might prefer to work alone to avoid sharing credit with others who contribute less, or not at all, to the paper, which is another form of escaping strategy. In short, the dilution, downgrading and homogenization effects in multi-authored papers might lead to escaping strategies, which may, in turn, favor SPs.

However, the well-established long-term trend toward team publication to the detriment of SPs (Fig. 1) suggests the opposite. This can be explained, in part, by a relatively recent practice: the last author is implicitly recognized as the team leader, the grant holder, the project manager or the head of the laboratory. This individual does not necessarily participate in the actual research or in writing the paper—tasks that are typically carried out by graduate students or postdoctoral researchers, who usually appear as first authors. For this reason, the terms junior author and senior author have become common in multi-authored papers to refer to the first and last authors, respectively. It is usually assumed that the junior author conducted most of the work, while the senior author provided the intellectual leadership.

Initiatives such as the Contributor Roles Taxonomy (CRediT) aim to clarify this issue (McNutt et al, 2018) and are now widely used by leading journals and publishing houses. However, this practice relies on what coauthors report about their respective roles in a given paper, which is highly subjective, and is often overestimated (Herz et al, 2020). In addition, within this approach, the same credit may be given to a contributor who provides a reagent as to one who conceives the idea behind an important discovery (Rull, 2025). This failure to properly recognize credit allocation also has a downgrading effect that mainly favors intermediate coauthors who rarely, if ever, publish solo papers and are seldom first or last authors—the flocking-sheep strategy—and it penalizes those who do follow these practices, thereby encouraging escaping strategies.

Therefore, one reason behind the decline of SPs and the proliferation of team papers may be the increase in flocking-sheep practices, in which many authors who never or almost never publish SPs or take a leading role in multi-authored papers are encouraged. This also favors the so-called publication gangs, or publication cartels, in which the same team of authors produces many papers with rotating authorship patterns, and cite each other’s publications (Rull, 2025). Ultimately, the flocking-sheep strategy does not help eradicate authorship misconduct, notably guest or honorary authorship.

## Survival

In such an environment, why do SPs still survive? Or, in other words, why do some authors persist in publishing SPs? The answer seems mainly tied to proper credit allocation and personal recognition (Praus, 2025). A SP gives all credit unequivocally to the single author, which avoids the credit-homogenization effect. In this way, the author’s work is properly recognized and his/her reputation and prestige are protected. In times of strong dominance of collaborative papers, this lone-wolf or escaping option is needed to secure and maintain an author’s individuality and distinctiveness. SPs are also a way for young scientists—whose contributions in collaborative research teams may be overlooked—to gain recognition in the early stages of their careers as independent researchers.

“In times of strong dominance of collaborative papers, this lone-wolf or escaping option is needed to secure and maintain an author’s individuality and distinctiveness.”

Another motive for publishing SPs may be the quest for intellectual influence, especially when an author identifies topics or ideas that are insufficiently developed—or not developed at all—and wishes to highlight their usefulness for scientific progress. As suggested above, this requires high levels of personal and professional independence and is difficult to achieve when collaboration demands theoretical and conceptual agreement. The same body of evidence may be interpreted differently by different scholars, and minimum agreements are usually subject to the downgrading effect. Discussions in journal forums are more useful for bringing ideas to light and avoiding consensus-driven trimming. This practice is more useful for readers, as it allows them to form their own opinions on the subject, thereby promoting theoretical and conceptual reflection.

Writing books is another task usually accomplished by individual authors. Most meta-analyses on publication trends and patterns use general databases (WOS, Scopus) that include only journal papers. However, synthesizing knowledge in a book is also a way of influencing scientific trajectories. The lack of comprehensive, standardized and universally accepted indexing systems and quantitative measures of book impact comparable to those available for journals makes this influence more difficult to analyze objectively. It is often assumed that major and well-known academic publishing companies produce books of higher quality, but this is difficult to test with the available information. Nevertheless, any scientist knows which books are the most influential in their field of research, and most of them were written by single authors.

For example, as a biologist working on evolution, one of the most influential books I know is Ernst Mayr’s (2004) *What Makes Biology Unique?*, which marked a before-and-after moment in biological thinking. Mayr (1904–2005) was a prolific evolutionary scientist with a high percentage of single-author publications, especially books. However, his personal influence is universally recognized and does not require impact metrics to be acknowledged. Once more, not all authors of SPs are like Mayr, but the emphasis here is on authorship patterns. I am sure that this—a highly influential book written by a single author—is the case in many scientific fields.

## Final remarks

Hallock (2008) emphasizes that publishing SPs requires intellectual independence (I) and tenacity (T), two qualities desirable in any researcher. I would add creativity (C) and the capacity for complex (X) and abstract (A) thinking, to form the TAXIC pack. In general, lone-wolf and escaping strategies require high TAXIC levels, whereas the flocking-sheep strategy is less demanding and easier to adopt, especially for authors contributing to high-impact papers based on others’ work and ideas. The TAXIC pack is needed to synthesize knowledge for writing books and review papers that transcend the simple accumulation of facts and become useful state-of-the-art syntheses with novel proposals for future developments. It is also required to write impactful theoretical and conceptual contributions such as essays or opinion papers. Finally, it should be stressed that authors of SPs may also publish team papers, as these are not mutually exclusive tasks. My point is not that most publications should be single-authored, but that there is still a niche for them and their survival should be encouraged.

Unfortunately, the current institutional scientific environment is not well suited for SPs, given the dominant role of collaborative research teams (Adams, 2013). For example, in Europe, multinational macro-research initiatives aimed at providing practical solutions to immediate industrial and societal needs are explicitly prioritized for funding, which threatens scientific freedom (Rull, 2016). Under such politically biased conditions, a research project such as Darwin’s—5 years of field exploration aboard a ship, followed by individual data processing and culminating in a solo book—would never be funded. At present, no more Darwins are envisioned on the horizon, but the likelihood that similar geniuses might emerge is explicitly and implicitly constrained by the scientific system. It is also common for national and international evaluation and funding bodies to give more value to data papers than to theoretical and conceptual papers, which mostly fall within the domain of SPs. Given the relevance that SPs and solo books have, and have had, for scientific progress, they should nevertheless be encouraged within the global science system.

“At present, no more Darwins are envisioned on the horizon, but the likelihood that similar geniuses might emerge is explicitly and implicitly constrained by the scientific system.”

## Supplementary information

Peer Review File
