# Supplementary material for: Does solo publication still make sense? Single-authored publications have been essential to scientific progress but are now facing extinction
Source: EMBO Rep. 2025 Dec 16;27(3):566–9. doi: 10.1038/s44319-025-00677-1 (PMC12894964; doi:10.1038/s44319-025-00677-1)
Supplement: Supplementary file 1 — Peer Review File [file 44319_2025_677_MOESM1_ESM.pdf]

EMBO reports

Does solo publication still make sense?

Valentí Rull

Corresponding author(s): Valentí Rull (vrull@csic.es)

Review Timeline:

Submission Date:	16th Nov 25
Accepted:	8th Dec 25

Editor: Holger Breithaupt

Transaction Report: This article was reviewed by an editorial board member.
